# Utilisation and consequences of CRP point-of-care-testing in primary care practices: a real-world multicentre observational study with 1740 patient cases in Germany

**DOI:** 10.3399/BJGPO.2024.0120

**Published:** 2025-01-29

**Authors:** Robby Markwart, Lena-Sophie Lehmann, Markus Krause, Paul Jung, Liliana Rost, Susanne Doepfmer, Lisa Kuempel, Doreen Kuschick, Kahina J Toutaoui, Christoph Heintze, Jutta Bleidorn, Florian Wolf

**Affiliations:** 1 Institute of General Practice and Family Medicine, Jena University Hospital, Friedrich Schiller University, Jena, Germany; 2 InfectoGnostics Research Campus Jena, Jena, Germany; 3 Charité – Universitätsmedizin Berlin, corporate member of Freie Universität Berlin and Humboldt-Universität zu Berlin, Institute of General Practice, Berlin, Germany

**Keywords:** diagnosis, infectious illness, family medicine, C-reactive protein, point-of-care testing, general practitioners, primary healthcare

## Abstract

**Background:**

C-reactive protein point-of-care tests (CRP-POCTs) can support GPs' clinical decision making but they are not widely used in German general practices.

**Aim:**

To investigate the utilisation of semi-quantitative CRP-POCTs in routine primary care.

**Design & setting:**

Prospective observational study in 49 general practices in Germany (from November 2022–April 2023).

**Method:**

GPs were provided with CRP-POCTs and collected data for each CRP-POCT use, with standardised data-collection sheets.

**Results:**

Data from 1740 CRP-POCT uses were recorded. GPs employed CRP-POCTs mainly for patients with respiratory tract infections (RTIs; 71.2% of all cases) and to a lesser extent for gastrointestinal infections (GIs; 10.4%). In RTIs, CRP-POCTs were frequently used to distinguish between bacterial and viral aetiology (60.8%) and to guide decisions on antibiotic prescribing (62.8%). In GIs, CRP-POCTs were mainly used to rule out severe disease progressions (53.2%) and for decisions on further diagnostic procedures (45.6%). In RTIs, CRP-POCTs influenced antibiotic prescribing in 77.5% of the cases (32.3% in favour versus 45.2% waiver). In GIs, CRP levels mainly affected decisions on further diagnostic procedures. GPs reported that CRP-POCTs were helpful in 88.6% of all cases.

**Conclusion:**

When available, German GPs predominantly use semi-quantitative CRP-POCTs to guide decisions on antibiotic prescribing in patients with RTIs. CRP-POCT use improves clinical decision making and increases the GP’s clinical confidence.

## How this fits in

Although C-reactive protein point-of-care tests (CRP-POCTs) are widely used for patients with signs of infection in many countries, they are not commonly used in the UK or German general practices. In this prospective multicentre study, we provided 49 general practices with semi-quantitative CRP-POCTs, and data were collected from 1740 patients on whom CRP-POCTs were used. This study presents data on the indications, diagnostic goals, clinical consequences of CRP-POCT use, and its impact on GPs’ clinical confidence and patient communication. In contrast to many other studies, our study is not limited to the effects of CRP-POCTs on antibiotic prescribing for patients with respiratory tract infections (RTIs).

## Introduction

Point-of-care tests (POCTs) for C-reactive protein (CRP) can support GPs' clinical decision making during the patient visit. CRP is an acute-phase protein secreted mainly by the liver within 24 hours in response to inflammation and injury. Measurements of CRP can help to differentiate between acute bacterial and viral infections, although its diagnostic accuracy for detecting of bacterial infections may be limited.^1,2^ In addition, CRP values provide information on the severity of inflammatory processes and can be used to monitor the effectiveness of antibiotic therapies.^3–5^

Multiple studies have shown that the use of CRP-POCTs for RTIs can safely reduce antibiotic prescribing in primary care settings.^3,6–8^ Owing to their diagnostic value in primary care, CRP-POCTs have been implemented in many European countries, such as Denmark, The Netherlands, Norway, Sweden, and Switzerland.^9–12^ However, in other countries, such as the UK^11,13^ and Germany,^14^ CRP-POCTs are not frequently used in routine primary care. In Germany, CRP-POCTs are employed by <20% of GPs,^14,15^ which may be explained by the low reimbursement (1.15 Euros [approximately 0.96 GBP] per test for patients with statutory health insurance) in relation to purchasing costs (2–4 Euros [approximately 1.70–3.40 GBP]).

Very few studies have investigated the clinical indications, reasons, consequences, and diagnostic value of CRP-POC testing in German general practices.^16^ The aim of this study was to investigate the utilisation of semi-quantitative CRP-POCTs in German general practices, including the reasons for performing CRP-POCTs, clinical indications, the effects on clinical decision making as well as perceived usefulness.

## Method

This study followed the Strengthening the Reporting of Observational Studies in Epidemiology (STROBE) statement: guidelines for reporting observational studies.^17^

### Study design

We conducted a prospective multicentre cross-sectional observational study in 49 general practices in three federal states in Eastern Germany (Berlin, Brandenburg, and Thuringia) from November 2022–April 2023. Outpatient physicians, including GPs, from the study region are characterised by below-average antibiotic prescribing rates compared with physicians in other regions of Germany, especially with those in South and Southwest Germany.^18^ General practices were recruited from members of the Research Practice Network East (RESPoNsE)^19^ using a purposive sampling to achieve an even distribution between Thuringian practices and practices from Berlin and Brandenburg. Furthermore, the sample of general practices was intended to reflect the distribution of different practice types and locations of the total general practice population in these regions. Each participating general practice was provided with 50 semi-quantitative CRP-POCTs, including lancets (RightSign, sensitivity: 99.4%, specificity: 97.1%, as reported by the manufacturer). The semi-quantitative CRP-POCT used is based on a lateral flow assay and the range of CRP levels is indicated by the number of test lines (no test line: CRP<10 mg/l, one test line: CRP 10–39 mg/l, two: CRP 40–80mg/l, three: CRP>80 mg/l). Semi-quantitative CRP-POCTs were chosen over quantitative CRP-POCT systems because they are easier to implement in German general practices, as they require neither an expensive analyser platform nor an extensive training of the practice staff. Furthermore, since the COVID-19-pandemic, lateral flow assays have been well known to the public. In addition to the CRP-POCTs, GPs were provided with a one-page document on the study procedure and manufacturer’s information on the CRP-POCTs. The study researchers did not provide any guidance or restrictions on when to use or not use the tests. GPs were free to decide on the use of CRP-POCTs, including use in the general practice, and also in other primary care settings such as home visits and/or out-of-hours emergency medical services.

All patients seen by the GPs during the study period were eligible, regardless of age, sex, (suspected) disease, comorbidities, and so on. We considered five domains (eligibility, recruitment, setting, organisation, and flexibility or delivery) from the PRECIS-2-PS tool to ensure the most pragmatic 'usual care conditions' possible.^20^

### Data collection and statistical analyses

GPs were requested to complete standardised data-collection sheets (see Supplementary Information) for each patient on whom they performed a CRP-POCT. As a literature search did not identify a validated questionnaire suitable for our objectives, we developed our own data-collection sheet based on our previous studies.^21,22^ The data-collection sheet was piloted by GPs before the study. Only anonymous patient data were collected. Participants received financial compensation based on the number of completed and returned data-collection sheets.

Raw data from the data-collection sheets were entered into Microsoft Excel 2010. Descriptive statistical analyses and data visualisation were performed using R (version 4.2.2)^23^ and RStudio (version 2022-12-03).^24^ In order to investigate the statistical relationships between the CRP concentration and the decisions made by GPs, Pearson’s χ^2^ test with Yates' continuity correction was employed.

In addition to the study described here, we conducted semi-structured interviews with a subset of the participating GPs to investigate the experiences, barriers, and facilitators to CRP-POCT use in German general practices. The results of this qualitative study will be published in a separate article.

## Results

### Characteristics of participating general practices and included patients

Forty-nine general practices participated in this study (Table 1). During the six-month study period, GPs performed CRP-POCTs on 1740 patients and returned the corresponding data-collection sheets, defined as patient cases. On average (median), each general practice included 44 patient cases. The majority of patients were female (62.2%) and the median age was 50 years. The majority of practices (57.1%) were solo practices. Group practices accounted for 32.7% and ambulatory healthcare centres for 10.2% of the included practices.

**Table 1.** Characteristics of participating general practices and included patient cases

Characteristic	*n*	(%)
**Number of participating general practice**	49	(100%)
**Practice type (*n*, %**)		
Solo practices	28	(57.1%)
Group practices	16	(32.7%)
Ambulatory healthcare centres	5	(10.2%)
**Median number of employed healthcare workers per practices (IQR)**		
GPs, including doctors in vocational training	2	(1–3)
Medical assistants	3	(2–5)
**Practice location (German federal state)*, n* (%**)		
Berlin	12	(24.5%)
Brandenburg	13	(26.5%)
Thuringia	24	(49.0%)
**Size of practice location^a^ (%**)		
Large urban centre (≥500 000 pop.)	12	(24.5%)
Urban centre (100 000–499 999 pop.)	13	(26.5%)
Large town (20 000–99 999 pop.)	12	(24.5%)
Small town (5000–19 999 pop.)	5	(10.2%)
Rural community (<5000 pop.)	7	(14.3%)
**Median patient cases^a^ (IQR)**	1740	(100%)
**Median number of patient cases per practices (IQR**)	44	**(23–48)**
**Median patient age (IQR**)	50	(34–63)
**Patient’s sex,** ^b^ ***n* (%**)		
Female	1072	(62.2%)
Male	652	(37.8%)
*Diverse/unknown*	*16*	*-*
**Diagnosis (suspected or confirmed),** ^b^ ***n* (%)**		
Respiratory tract infection	1195	(71.2%)
Gastrointestinal infection	174	(10.4%)
Urinary tract infection	96	(5.7%)
Skin and soft tissue infections	82	(4.9%)
Unspecified infection	82	(4.9%)
Other	49	(2.9%)
*Unknown*	*62*	*-*
**SARS-CoV-2 status,^b^ , *n* (%)**		
No evidence for SARS-CoV-2 infection	1514	(94.8%)
Relevant evidence for SARS-CoV-2 infection (symptoms+contact to SARS-CoV-2 patient)	51	(3.2%)
Proven SARS-CoV-2 infection (positive SARS-CoV-2 rapid-antigen test or PCR test within last 7 days)	32	(2.0%)
*Unknown*	*143*	*-*
**CRP-level measured,** ^**b**^ ***n*** **(%)**		
Negative (<10 mg/l)	851	(49.6%)
Slightly increased (10–39 mg/l)	283	(16.5%)
Moderately increased (40–80 mg/l)	360	(21.0%)
Strongly increased (>80 mg/l)	220	(12.8%)
*Not specified*	*26*	*-*

^a^Number of performed CRP-POCTs and returned data-collection sheets. ^b^Percentage among valid answers (exclusion of unknown). CRP-POCTs = C-reactive protein point-of-care tests. IQR = interquartile range. PCR = polymerase chain reaction

### Clinical indications and diagnostic goals for CRP-POCT use

GPs mainly used CRP-POCTs on patients with symptoms of respiratory tract infections (RTIs; 71.2% of all cases). Other clinical indications were evidence of gastrointestinal infections (GIs; 10.3%), urinary tract infections (UTIs; 5.7%), skin and soft tissue infections (SSTIs; 4.9%), and unspecified infections (4.9%) (Table 1).

In approximately half of the patients, GPs performed CRP-POCTs to decide whether antibiotic treatment was necessary (55.0%) and to differentiate between bacterial and viral infections (48.0%). In addition, CRP-POCTs were frequently used to rule out severe diseases and/or disease progressions (40.3%). Conversely, GPs used CRP-POCTs less frequently to determine the need for further diagnostics (23.0%), hospital admissions (6.8%), and monitoring purposes such as therapy success (6.5%).

**Figure 1.**
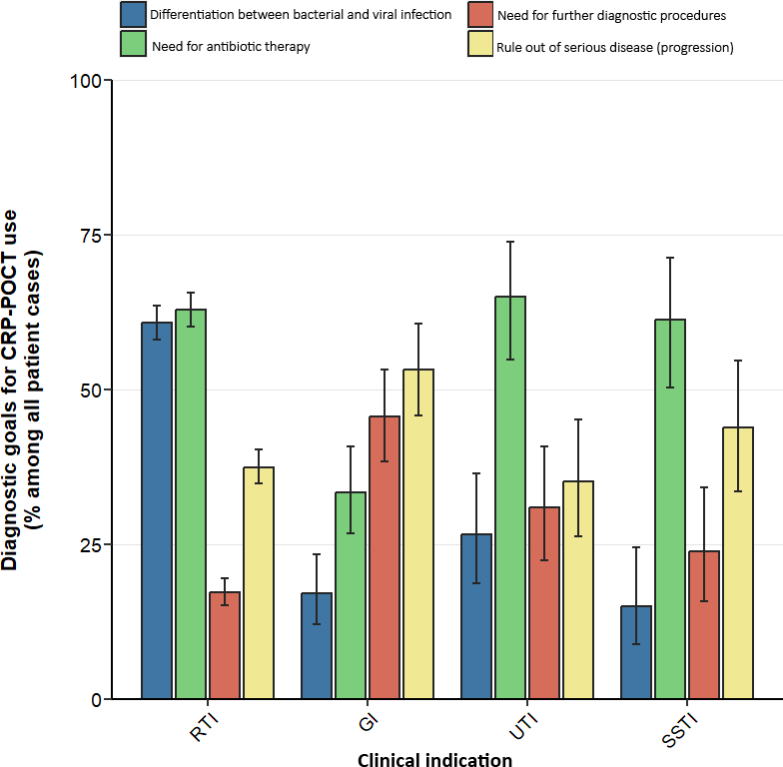
Diagnostic goals for the use of semi-quantitative CRP-POCTs by German GPs. Diagnostic goals as reported by GPs displayed as percentage (%) among all valid answers stratified by clinical indication. RTI = respiratory tract infection (*n* = 1173). GI = gastrointestinal infection (*n* = 171). UTI = urinary tract infection (*n* = 94). SSTI = skin and soft tissue infection (*n* = 80). Error bars indicate 95% confidence intervals.

The diagnostic goal for CRP-POCT utilisation differed between clinical indications. In patients with (suspected) RTI, GPs frequently employed CRP levels to distinguish between bacterial and viral aetiology (60.8%) (Figure 1). CRP-POCTs were often used to guide decisions on antibiotic prescribing in RTIs (62.8%), UTIs (64.9%), and SSTIs (61.3%), but less frequently in GIs (33.3%). In GIs, CRP-POCTs were most frequently used to rule out severe diseases and/or disease progressions (53.2%) as well as for the decision on the need for additional diagnostic procedures (45.6%).

### CRP test results and clinical consequences

In almost half of all patient cases (49.6%) the CRP-POCT test result was negative (that is <10 mg/l), while in 21.0% of patients CRP levels were increased moderately (40–80 mg/l) and in 12.8% strongly (>80 mg/l) (Table 1).

In over two-thirds of all patient cases (69.1%), the use of CRP-POCTs influenced the GP’s clinical decision and in an additional 13.4% of the cases, CRP levels impacted clinical decision making at least partly.

In about two of three patient cases (67.3%), the use of CRP-POCTs influenced the decision on antibiotic prescribing, with a greater proportion of decisions made against antibiotic prescribing (38.7%) compared with those in favour of antibiotic therapy (28.6%) (Table 2). The decisions of GPs regarding antibiotic prescribing were found to be significantly (*P* value <0.001) influenced by CRP test results. In cases where the CRP value was moderately or strongly increased (≥40 mg/l), GPs were more likely to prescribe antibiotics (65.6% of cases), while in cases where the CRP value was low (<40 mg/l), GPs were less likely to prescribe antibiotics (9.9% of cases).

**Table 2.** Reported consequences of semi-quantitative CRP-POCT utilisation on clinical decisions, stratified by CRP values

	All cases (*n* = 1701)		CRP<40 mg/l (*n* = 1114)		CRP≥40 mg/l (*n* = 569)		CRP<40 mg/l versus CRP≥40 mg/l
	*In favour* *‘yes’*	*Waiver ‘no’*	*In favour* *‘yes’*	*Waiver ‘no’*	*In favour* *‘yes’*	*Waiver ‘no’*	*P value (χ^2^)*
**Antibiotic prescribing**	487 (28.6%)	658 (38.7%)	110 (9.9%)	629 (56.5%)	373 (65.6%)	21 (3.7%)	<0.001^a^ (665.6)
**Further diagnostics**	317 (18.6%)	341 (20.0%)	142 (12.7%)	312 (28.0%)	172 (30.2%)	26 (4.6%)	<0.001^a^ (168.4)
**Hospital admission**	52 (3.1%)	103 (6.1%)	7 (0.6%)	85 (7.6%)	44 (7.7%)	17 (3.0%)	<0.001^a^ (65.8)

^a^The Pearson’s χ^2^ test with Yates' continuity correction was used to compare consequences of CRP-POCT use between patient cases with CRP<40 mg/l and those with CRP≥40 mg/l. CRP-POCTs = C-reactive protein point-of-care tests

In 38.6% of the patient cases, CRP levels had an impact on decisions regarding additional necessary diagnostic procedures (18.6% in favour of additional diagnostic procedures, 20.0% no additional diagnostic procedures needed). Less frequently, GPs reported that the use of CRP-POCTs improved the communication with patients (18.2%) and influenced decisions on hospital admission (9.2%).

**Figure 2.**
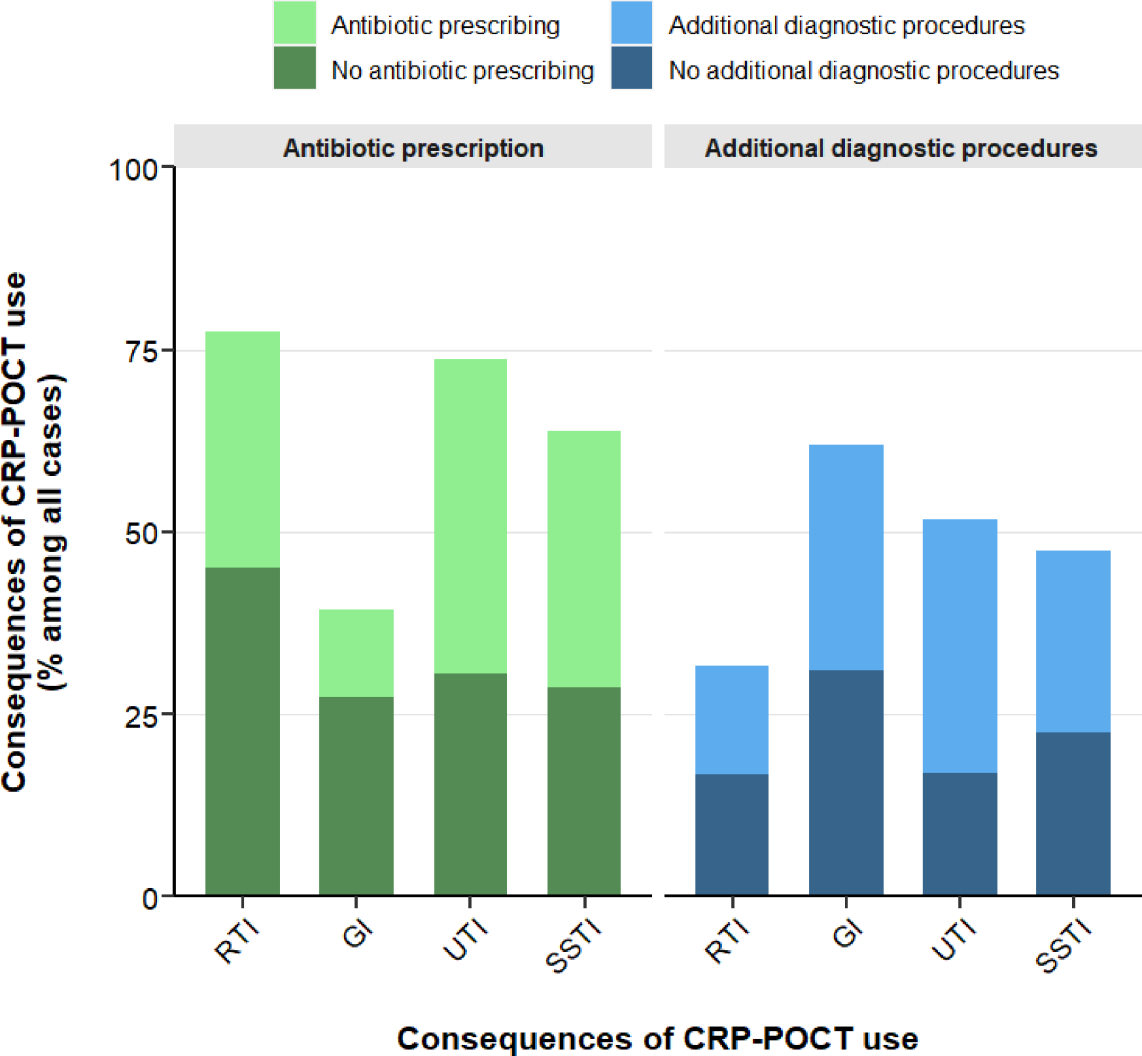
Reported consequences of semi-quantitative CRP-POCT utilisation by clinical indication. Consequences of CRP-POCT use regarding antibiotic prescribing and the need for additional diagnostic procedures as reported by GPs displayed as percentage (%) among all valid answers stratified by clinical indication. RTI = respiratory tract infection (*n* = 1178). GI = gastrointestinal infection (*n* = 168). UTI = urinary tract infection (*n* = 95). SSTI = skin and soft tissue infection (*n* = 80).

Reported consequences owing to CRP-POCT use on clinical decision making differed between clinical indications (Figure 2). Antibiotic prescribing behaviour was frequently influenced by CRP levels in RTIs, UTIs, and SSTIs, whereas the need for additional diagnostic procedures was more frequently influenced by CRP-POCTs in GIs. It is noteworthy that GPs reported that in UTI and SSTI, CRP-POCT use more frequently influenced decisions in favour of antibiotic prescribing compared with decisions against antibiotics.

### The impact of CRP-POCTs on clinical confidence and perceived usefulness

In most patient cases (83.7%), the use of CRP-POCTs increased the GP’s confidence in the treatment situation, and in 10% of the cases, the confidence was increased at least partly by CRP measurements. Moreover, GPs reported that CRP-POCTs were helpful (88.6%) or at least partly helpful (9.0%) in nearly all cases. The impact of CRP-POCT use on clinical confidence and the reported helpfulness was similar between patients with RTI and non-RTI-related diseases.

## Discussion

### Summary

In this study, we analysed 1740 cases of patients to whom semi-quantitative CRP-POCTs were performed by German GPs as part of their routine care. As expected, owing to the survey period (November 2022–April 2023), which is the typical cold and flu season in Germany, CRP-POCTs were primarily employed for patients exhibiting symptoms of RTIs. To a lesser extent, CRP-POCTs were also used for suspected infections of the urinary and gastrointestinal tract and of the skin and soft tissue. German GPs commonly employed CRP-POCTs to guide decisions on antibiotic prescribing and to rule out severe diseases and disease progressions. GPs' clinical decisions were impacted by CRP levels in almost all instances, specifically with regard to antibiotic prescription and the necessity for additional diagnostic tests. GPs reported that the use of CRP-POCTs enhanced their clinical confidence and were considered as overall helpful.

### Strength and limitations

To our knowledge, our multicentre prospective study is the first evaluation on the utilisation of CRP-POCTs in German general practices with focus on clinical indications, diagnostic goals, and the impact on GPs’ clinical decision making. The major strengths of this study are the pragmatic research approach, the large number of included patient cases and participating general practices in three different federal states of Germany.

However, our study has several limitations. First, the recruitment of general practices from a practice-based research network led to a selection bias towards GPs interested in research, which may differ from the total GP population in Germany. However, practice types and sizes of practice location of our sample were similar to those of the general practices in the three federal states included in the study. Furthermore, as participation was voluntary, we cannot exclude a selection bias towards general practices with a positive attitude towards CRP testing. Second, we only analysed patient cases in which CRP-POCTs were actually performed, which also introduced a bias towards cases in which GPs may have had a diagnostic uncertainty and therefore found CRP-POCTs helpful. Third, we did not collect clinical data from patients, so we could not determine the clinical accuracy of any clinical decision based on CRP testing. Finally, we were not able to record all cases of acute infections treated during the study period, so we were not able to calculate the frequency of CRP-POCT use.

### Comparison with existing literature

Our findings show that German GPs mainly use CRP-POCTs in patients with clinical signs for RTI in order to determine the need for antibiotics and to differentiate between bacterial and viral infections. This application of CRP-POCTs is in accordance with various German guidelines recommending CRP measurements to guide antibiotic therapy in patients presenting with signs of RTIs.^25–28^ In our study, GPs reported that the use of CRP-POCTs resulted in a greater proportion of decisions made against antibiotic prescribing compared with those in favour of antibiotic therapy. This aligns with existing evidence on the effectiveness of CRP-POCTs in promoting rational antibiotic prescribing in RTIs in primary care.^3,6–8,29^ It is important to note that CRP thresholds for guiding antibiotic prescribing vary across studies and clinical guidelines.^10^ This makes the interpretation of CRP test results challenging for GPs. In the UK and The Netherlands, evidence-based guidelines recommend antibiotic therapy if the CRP concentration is greater than 100 mg/l, while antibiotic therapy should not be considered as a routine option if CRP values are <20 mg/l. A delayed antibiotic prescribing strategy is recommended for CRP values between 20 mg/l and 200 mg/l.^30,31^

Although participating GPs reported that semi-quantitative CRP-POCTs were helpful in most cases and increased their clinical confidence, other studies have described several limitations of CRP-POCTs. CRP, as a biomarker for detecting bacterial infections has a limited diagnostic accuracy^1,2^ and semi-quantitative CRP-POCTs often have a poor diagnostic performance.^32^ Moreover, the narrow time frame for visual test interpretation (5–8 minutes) and difficulties of users in evaluating of the line pattern have also been described as limitations of semi-quantitative CRP-POCTs.^33^

Based on these limitations, semi-quantitative CRP-POC testing should not be used as a sole diagnostic criterion for clinical decisions. CRP levels should always be interpreted in the context of other factors, including clinical symptoms, anamnesis, patient characteristics (for example, age) and risk factors for severe disease progressions. Clinical scores, such as the Centor and McIsaac scores for group A β-haemolytic streptococci pharyngitis, have been established to support diagnosis of bacterial upper RTIs, but also have a limited diagnostic accuracy.^34,35^ The combination of clinical scores and CRP-POC testing may offer a valid and accurate diagnostic algorithm to guide antibiotic prescribing in RTIs.

In our study, GIs accounted for 10.4% of CRP-POCT applications by GPs. However, CRP has an ambivalent diagnostic value in inflammatory diseases of the gastrointestinal tract. While CRP is an established marker for the diagnosis and prognosis of acute pancreatitis^36,37^ and diverticulitis,^38,39^ it possesses only a limited accuracy for diagnosing appendicitis.^40–42^

Our results show that CRP-POCTs enhanced the GP’s confidence in the treatment situation — also in non-RTI-related consultations — and were found to be helpful in almost all patient cases. Very similar results are reported from German physicians working in out-of-hours primary care^21^ and from general practices in England.^13^ Despite the positive attitude towards CRP-POCT and evidence on its diagnostic value in primary care, <20% of German GPs utilise CRP-POCTs.^14,15^ In contrast, GPs in other European countries tend to more commonly employ CRP-POCTs for acute infections.^7,9,10,12,37,43^ Similar to the UK, the relatively low uptake of CRP-POCTs in Germany can be attributed to time constraints, effects on clinical workflow, and costs.^13,14^ The reimbursement rate of 1.15 Euro per test for CRP-POCTs conducted by GPs in Germany does not even cover the purchase costs. However, our study suggests, that the deliberated and targeted use of CRP-POCT can be of benefit to GPs in routine medical care.

### Implications for practice

When available, German GPs predominantly use CRP-POCTs in patients with clinical signs of RTIs to guide decisions on antibiotic prescribing. Using CRP-POCTs in routine primary care impacts clinical decision making regarding the use of antibiotics and the need for further diagnostic measures. In cases where CRP-POCTs are used, they increase GPs’ confidence and are perceived as helpful, not only in RTIs, but also in other inflammatory diseases. Owing to difficulties in usability and test interpretation as well as potentially limited diagnostic accuracy, CRP levels should not be used as the sole diagnostic criterion. Instead, they should always be employed in the context of clinical symptoms, anamnesis, patient characteristics and risk factors for severe disease progression. Interventional studies, preferably randomised controlled trials, are needed to show the effectiveness and safety of CRP-POCT on patient health outcomes in German primary care.

## Trial registration number

The study protocol was published at the German Clinical Trials Register (registration number: DRKS00030333).

## Data

The dataset relied on in this article is available from the corresponding author on reasonable request.
